# Biomechanical comparison of menisci from different species and artificial constructs

**DOI:** 10.1186/1471-2474-14-324

**Published:** 2013-11-17

**Authors:** Gunther H Sandmann, Christopher Adamczyk, Eduardo Grande Garcia, Stefan Doebele, Andreas Buettner, Stefan Milz, Andreas B Imhoff, Stefan Vogt, Rainer Burgkart, Thomas Tischer

**Affiliations:** 1 Department of Orthopaedic Sport Medicine, Technische Universitaet Munich, Munich, Germany; 2 Department of Trauma Surgery, Technische Universitaet, Munich, Germany; 3 Department of Neurology, Ludwig- Maximilians- Universitaet Munich, Munich, Germany; 4 Division of Biomechanics, Department of Orthopaedic Surgery, Technische Universitaet Munich, Munich, Germany; 5 Department of Forensic Medicine, University of Rostock, Rostock, Germany; 6 Department of Anatomy, Ludwig- Maximilians- Universitaet Munich, Munich, Germany; 7 Department of Orthopaedic Surgery, University of Rostock, Doberanerstr. 142, Rostock D-18057, Germany

**Keywords:** Meniscus, Biomechanics, Animal model, Meniscus scaffolds

## Abstract

**Background:**

Loss of meniscal tissue is correlated with early osteoarthritis but few data exist regarding detailed biomechanical properties (e.g. viscoelastic behavior) of menisci in different species commonly used as animal models. The purpose of the current study was to biomechanically characterize bovine, ovine, and porcine menisci (each n = 6, midpart of the medial meniscus) and compare their properties to that of normal and degenerated human menisci (n = 6) and two commercially available artificial scaffolds (each n = 3).

**Methods:**

Samples were tested in a cyclic, minimally constraint compression–relaxation test with a universal testing machine allowing the characterization of the viscoelastic properties including stiffness, residual force and relative sample compression. T-tests were used to compare the biomechanical parameters of all samples. Significance level was set at p < 0.05.

**Results:**

Throughout cyclic testing stiffness, residual force and relative sample compression increased significantly (p < 0.05) in all tested meniscus samples. From the tested animal meniscus samples the ovine menisci showed the highest biomechanical similarity to human menisci in terms of stiffness (human: 8.54 N/mm ± 1.87, cycle 1; ovine: 11.24 N/mm ± 2.36, cycle 1, p = 0.0528), residual force (human: 2.99 N ± 0.63, cycle 1 vs. ovine 3.24 N ± 0.13, cycle 1, p = 0.364) and relative sample compression (human 19.92% ± 0.63, cycle 1 vs. 18.72% ± 1.84 in ovine samples at cycle 1, p = 0.162). The artificial constructs -as hypothesized- revealed statistically significant inferior biomechanical properties.

**Conclusions:**

For future research the use of ovine meniscus would be desirable showing the highest biomechanical similarities to human meniscus tissue. The significantly different biomechanical properties of the artificial scaffolds highlight the necessity of cellular ingrowth and formation of extracellular matrix to gain viscoelastic properties. As a consequence, a period of unloading (at least partial weight bearing) is necessary, until the remodeling process in the scaffold is sufficient to withstand forces during weight bearing.

## Background

The important role of the meniscus in load transmission and shock absorption is well known [1,2]. It is obvious that loss of meniscus tissue leads to focal overload of articular cartilage and finally to a higher incidence of osteoarthritis [1,3]. Unfortunately, meniscus tears are very common and (partial) resection of the damaged tissue is necessary in many cases [3,4]. To avoid the negative side effects of (total or partial) meniscectomy [5,6], alternative treatment options, especially for young individuals, are required. Meniscus allograft transplantation has been reported to reduce pain and improve knee joint function [7]. However, there are still problems like the sizing of implants, risk of disease transmission, and limited availability [8,9]. In addition, degeneration, tears and structural alterations were noticed throughout the remodeling process in several studies dealing with early meniscus allograft transplantation [10,11].

Alternatively, artificial scaffolds such as Menaflex (formerly CMI) or Actifit can be implanted. Menaflex is derived from devitalized bovine collagen (Achilles tendon) and this scaffold allows cellular ingrowth and improves tissue regeneration [10,12]. The use of Bone marrow derived stem cells under perfusion and cyclic compression has been shown to have a positive effect on the cellular ingrowth in vitro [13]. So far, several studies have proven the positive effect of Menaflex implantation on the clinical outcome measured by IKDC, Tegner Score and VAS [14-16]. Actifit is a polyurethane polymer scaffold and good biocompatibility and cellular ingrowth have been reported leading to an improvement of the frictional properties as reported by Galley et al. [17]. Nevertheless, the long-term benefits of these scaffolds in terms of chondroprotection are still a matter of debate [18], although mid-term results are promising [19,20]. Especially, studies about the biomechanical properties of theses polyurethane scaffolds at time of implantation are missing.

Whereas human meniscus has been characterized relatively well in several studies [21-23], biomechanical data of meniscus tissue of suitable animal models serving as a base for comparative studies are therefore of particular interest [24-26]. Sweigart et al. examined the biomechanical properties of baboon, bovine, canine, lapine, porcine and human meniscus samples [25]. They found significant variations in the material properties, which underline the problems in finding the perfect animal model and transferring the results to the human knee joint. As Chevrier et al. found the highest structural similarities in terms of vascularity and collagen structure between human and ovine menisci, our study aimed to close this gap [24].

Therefore, we characterized the biomechanical properties of different human (normal and degenerative) and animal (bovine, ovine, porcine) menisci and compared the results to two commercially available artificial scaffolds (Menaflex, Actifit). The midpart of the meniscus was chosen as it represents a well-defined region, which always can be identified and examined in all species irrespective of size differences.

## Methods

### Animal meniscus samples

Meniscus samples were harvested from fresh bovine (n = 6), ovine (n = 6) and porcine (n = 6) knee joints obtained from a local slaughterhouse. All animals were young at slaughter but skeletally mature with closed epiphyseal plates (bovine: average weight 135 kg, average age 7.3 months, ovine: average weight 85.2 kg, average age 12.1 months, porcine: average weight 92.5 kg, average age 11.3 months). Knees with macroscopic signs of degenerative or traumatic changes to the cartilage or obvious meniscal tears were excluded. Using a cylindrical device for “osteochondral autologous transplantation” (OATS donor device, Arthrex, Naples, Fl) three samples from the midpart (pars intermedia) of each medial meniscus, 8 mm in diameter and 4 mm in height were harvested. The meniscus specimens were stored in physiologic saline solution at 8°C until biomechanical testing (performed at room temperature). Testing was performed within 6 hours to avoid alterations due to prolonged storage.

### Human meniscus samples

Normal human meniscus samples were obtained from the Department of Forensic medicine and excluded if there were any signs of macroscopic degenerative knee disease or traumatic knee injury. Medial meniscus samples of 6 Caucasian male individuals with a mean age of 30.7 years (range 25-34 years, SD 3.27) were used. All had died due to diseases or accidents not affecting the knee. In contrast, the degenerative human meniscus samples were collected during operations with partial (n = 4) or total knee replacements (n = 2) with a mean patient age of 55.3 years (range 50-60 years, SD 3.67). In 2 patients the unicondylar prostheses became necessary due to partial meniscectomies in their twenties. The remaining four patients had posttraumatic arthritis after tibial head fractures explaining the relatively young age of our patients at time of prosthesis implantation. All human samples were treated as described above and three cylinders were harvested from each meniscus.

The study was approved by the Institutional Ethical Review Board of the Institute of Forensic Medicine. All patients approved the use of their tissues for this research project.

### Artificial constructs

The artificial scaffolds Menaflex (Regen Biologics, Hackensack, NJ, USA), formerly known as collagen meniscus implant (CMI), and Actifit (Orteq, London, UK) were used. From both scaffolds three samples (n = 3), were used to test their biomechanical properties. The samples were harvested with the OATS donor device (Arthrex, Naples, Fl), stored in physiologic saline solution for 10 minutes and kept moist throughout testing.

### Biomechanical characterization

**Figure 1:**
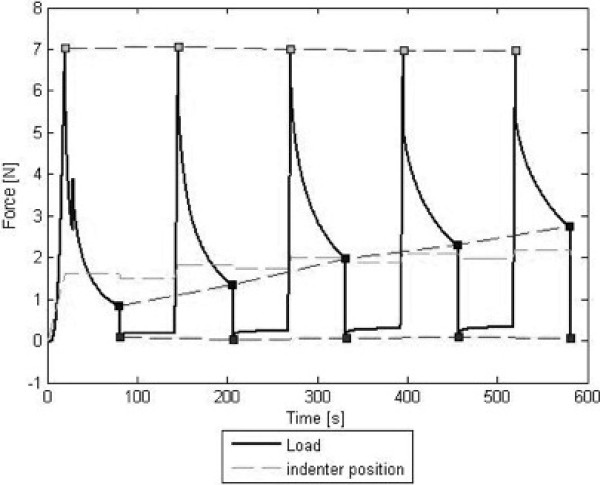
**Biomechanical graph showing the typical load curve of a test cycle consisting of five repetitive cycles showing the graphical course of preload, dynamic and static compression and relaxation.** Note the linear-elastic slope during dynamic compression.

To assess the biomechanical properties of human, animal and artificial samples a cyclic indentation test as minimally constraint compression-relaxation tests were performed [9]. Therefore a universal testing machine (Zwicki1120, Zwick, Ulm, Germany) with a calibrated depth and load sensitive indenter (KAP-S, A.S.T., Dresden, Germany) was used. The tip of the indenter consisted of a steel ball with a diameter of 5 mm. By using ball geometry for indentation, notch effects and stress concentrations (shear stress) at the contact area of the indenter with the specimens could be avoided. Such shear stresses would have been provoked using a flat indenter and tensile reactions would have possibly influenced our results. The meniscal specimens, trimmed to a rectangular shape, were placed horizontally under the testing device onto a specially designed smooth and flat metallic plate with a circular sink (diameter 10 mm, depth 0.2 mm) to achieve lateral stabilization during axial loading (minimally constraint to allow quasi-free evasion during testing). The indenter position was calibrated prior to each test; it was set to zero at the level of the base of the cavity. A preload of 0.5 N was applied to the specimens. The test-series comprised five repetitive indentation test-cycles consisting of the following phases: (1) Specimen preloading (0.5 N). (2) Dynamic compression of the sample with a constant load velocity of 5 mm/min until a peak test load of 7 N was achieved. (3) Static compression with the indenter remaining in the achieved position for 60 s. (4) Relaxation of the sample after 60 s with a constant velocity of 1 mm/min until a load of 0.1 N. The indenter remained in the achieved position for an interval of 60 s and the described test-cycle was then repeated another four times (Figure 1). Load, indenter position, and time were logged and displayed online by the test software package TestXpert (Version 8.1, Zwick). The stiffness (N/mm) of the meniscal specimens was determined from the linear-elastic slope of the loading curve between 2 N and 5 N. As parameters for the viscous properties we assessed the indentation depth (mm) and recorded the residual force (N) which was defined as the measured load at the end of the static compression phase. Biomechanical testing was performed at room temperature.

### Histology

For an overview of the principal tissue or scaffold organization, histologic sections were obtained from selected remaining tissue samples or scaffold material. After fixation of the human and animal meniscus samples in 90% methanol in 4°C for 48 h, specimens were infiltrated overnight in PBS with 5% sucrose at pH 7.4 and afterwards mounted on chucks in Jung tissue embedding medium (Leica, Germany), frozen in a HM 500 OMV cryostat (Mikrom, Germany) and cryosectioned at 12 μm. Sections were stained with hematoxylin and eosin and Safranin O staining. Artificial specimens were embedded in paraffin, sectioned and stained with Safranin O and light green. Safranin O staining highlights the accumulation of acidophil molecular complexes (proteoglycans and glycosaminoglycans) in the tissue.

### Statistics

Statistical analysis of the biomechanical data was performed using the software package SPSS (Version 11.0, SPSS Inc., Chicago, Illinois). After testing for normal distribution (Kolmogorov-Smirnov test) we used t-tests to compare the biomechanical parameters of animal, human and artificial meniscus samples. Significance level was defined at p < 0.05.

## Results

### Biomechanical results

The mean sample height was 3.9 mm (±0.2 mm) and all samples could be loaded up to 7 N without signs of plastic deformity. Slopes of the load curves were linear between 2 N and 5 N in all tests.

### Stiffness

**Figure 2:**
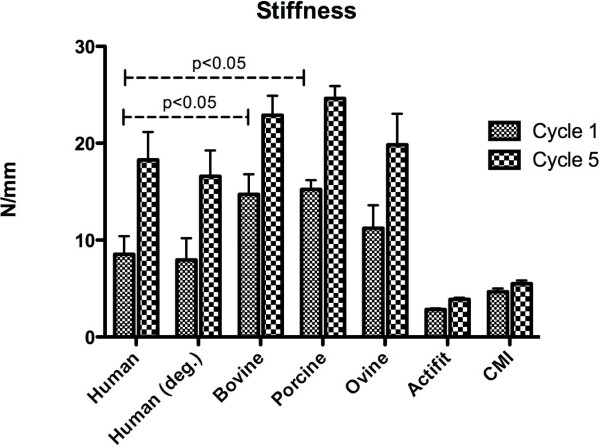
**Stiffness (N/mm) of all tested meniscus samples and the artificial scaffolds increased significantly between cycle 1 and 5.** P-values for statistically significant differences between human and porcine/ bovine meniscus samples. The difference between human and ovine samples was statistically not significant (p = 0.053 at cycle 1 and p = 0.399 at cycle 5), whereas the differences between the human meniscus and the synthetic constructs were statistically significant (p < 0.05).

The stiffness of all tested specimens increased significantly from cycle 1 to cycle 5 (p < 0.05, Figure 2). The initial stiffness of the normal human meniscus samples was 8.54 N/mm ± 1.87 (cycle 1) and increased throughout testing to 18.29 N/mm ± 2.88 (cycle 5; p = 0.003). Degenerative menisci showed slightly lower stiffness values: 7.94 N/mm ± 2.25 to 16.59 N/mm ± 2.66 (p = 0.007). All animal samples showed higher initial stiffness values: Bovine meniscus 14.72 N/mm ± 2.07 (cycle 1) to 22.89 N/mm ± 2.01 (cycle 5), p = 0.006; ovine samples 11.24 N/mm ± 2.36 to 19.84 N/mm ± 3.2 (p = 0.024); porcine meniscus 15.24 N/mm ± 0.95 to 24.63 N/mm ± 1.28 (p < 0.001). The artificial scaffolds were less stiff at baseline, but stiffness increased significantly as well: Actifit 2.83 N/mm ± 0.13 to 3.88 N/mm ± 0.17 (p = 0.013); Menaflex 4.66 N/mm ± 0.35 to 5.50 N/mm ± 0.33 (p = 0.039). The stiffness of bovine and porcine meniscus samples were significantly (p < 0.001) different from human meniscus tissue- only the ovine samples did not have statistically different stiffness values compared to human meniscus samples with p = 0.0528 (cycle 1) and p = 0.399 (cycle 5).

### Residual force

**Figure 3:**
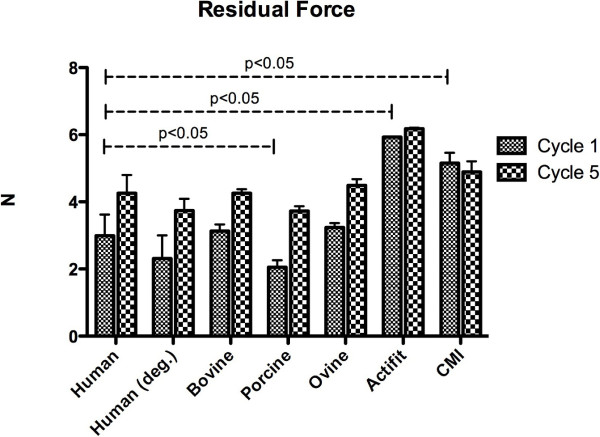
**Residual force (N) of the menisci and the scaffolds.** During testing the residual force increased in all tested samples. There were no statistical significant differences between human and ovine, respectively bovine samples (p > 0.05, each). However, the differences of residual force between human and porcine and human and synthetic constructs were statistically significant (p < 0.05).

The residual force of the normal and the degenerative human meniscus increased throughout testing (normal human meniscus: 2.99 N ± 0.63 to 4.26 N ± 0.54 (p = 0.0038) degenerative human meniscus: 2.31 N ± 0.69 to 3.74 N ± 0.35 (p = 0.0011; Figure 3). Similar to the changes in human meniscus samples, in ovine menisci the residual force increased from 3.24 N ± 0.13 in cycle 1 to 4.49 N ± 0.19 in cycle 5 (p < 0.001). The increase of the residual force in bovine (3.13 N ± 0.2 to 4.45 N ± 0.12, p < 0.001) and porcine meniscus samples (2.05 N ± 0.21 to 3.73 N ± 0.14) was also statistically significant (p < 0.001). The residual force of the Actifit showed a significant increase throughout testing (5.93 N ± 0.017 to 8.18 N ± 0.032, p < 0.001), whereas the Menaflex scaffold did not increase significantly (5.15 N ± 0.31 to 5.58 N ± 0.32, p = 0.265). There was no statistically significant difference concerning the residual force between normal human meniscus and all animal samples, except the residual force in human vs porcine samples at cycle 1 (p = 0.006). The artificial scaffolds Menaflex and Actifit were significantly different in their residual force, independently from the cycle (p < 0.05).

### Compression behavior

**Figure 4:**
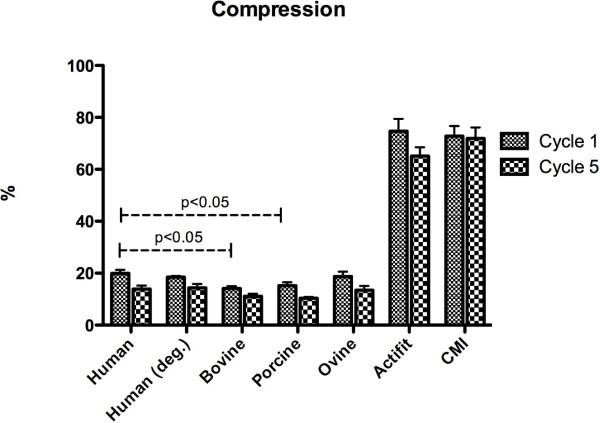
**Compression (%) of the menisci and the scaffold.** Note that the artificial scaffolds and the animal meniscus except the ovine samples showed significant differences compared to human samples. The differences between the human meniscus samples and the synthetic constructs were statistically highly significant (p < 0.0001).

After dynamic compression the mean compression of the normal human meniscus samples was 19.92% ± 1.36 (cycle 1) and 13.58% ± 1.33 (cycle 5) compared to 18.43% ± 055 (cycle 1) and 14.38% ± 1.14 (cycle 5) in degenerative meniscus samples (not statistically significant, p > 0.05, Figure 4). In the animal samples the compression of the ovine samples showed no statistically significant differences to human samples with 18.72% ± 1.84 (cycle 1) and 13.43% ± 1.61 (cycle 5); p > 0.05 each. Bovine and porcine samples showed statistically significant differences to normal human samples with 14.05% ± 0.92 (cycle 1, bovine; p < 0.0001), 11.10% ± 0.95 (cycle 5, bovine; p < 0.0001), respectively 15.21% ± 1.25 (cycle 1, porcine; p < 0.0001) and 10.31% ± 0.53 (cycle 5, porcine; p < 0.0001). Both artificial scaffolds Actifit and Menaflex showed statistically highly significantly different compression behavior compared to normal human and animal meniscus samples: Actifit: 74.7 ± 4.76 (cycle 1) and 65.1% ± 3.38 (cycle 5) with p < 0.0001 and Menaflex: 72.8% ± 3.86 (cycle 1), 71.89% ± 7.23 (cycle 5) with p < 0.05.

All relevant biomechanical parameters are visualized in Table 1.

**Table 1:** Summary of the biomechanical results showing the viscoelastic properties including stiffness, residual force and compression

	**Human (n = 6)**	**Human deg. (n = 6)**	**Bovine (n = 6)**	**Ovine (n = 6)**	**Porcine (n = 6)**	**Actifit (n = 3)**	**Menaflex (n = 3)**
Stiffness [N/mm], cycle1	8.54 ± 1.87	7.94 ± 2.25	14.72 ± 2.07	11.24 ± 2.36	15.24 ± 0.95	2.83 ± 0.13	4.66 ± 0.35
Stiffness [N/mm], cycle 5	18.29 ± 2.88	16.59 ± 2.66	22.89 ± 2.01	19.84 ± 3.2	24.63 ± 1.28	3.88 ± 0.17	5,50 ± 0.33
Resiudal Force [N], cycle 1	2.99 ± 0.63	2.31 ± 0.69	3.13 ± 0.2	3.24 ± 0.13	2.05 ± 0.21	5.93 ± 0.017	5.15 ± 0.31
Residual Force [N], cycle 5	4.26 ± 0.54	3.74 ± 0.35	4.45 ± 0.12	4.49 ± 0.19	3.73 ± 0.14	8.18 ± 0.032	5.58 ± 0.32
Compression [%], cycle 1	19.92 ± 1.36	18.43 ± 055	14.05 ± 0.92	18.72 ± 1.84	15.21 ± 1.25	74.7 ± 4.76	72.8 ± 3.86
Compresson [%], cycle 5	13.58 ± 1.33	14.38 ± 1.14	11.10 ± 0.95	13.43 ± 1.61	10.31 ± 0.53	65.1 ± 3.38	71.89 ± 7.23

### Histological results

**Figure 5:**
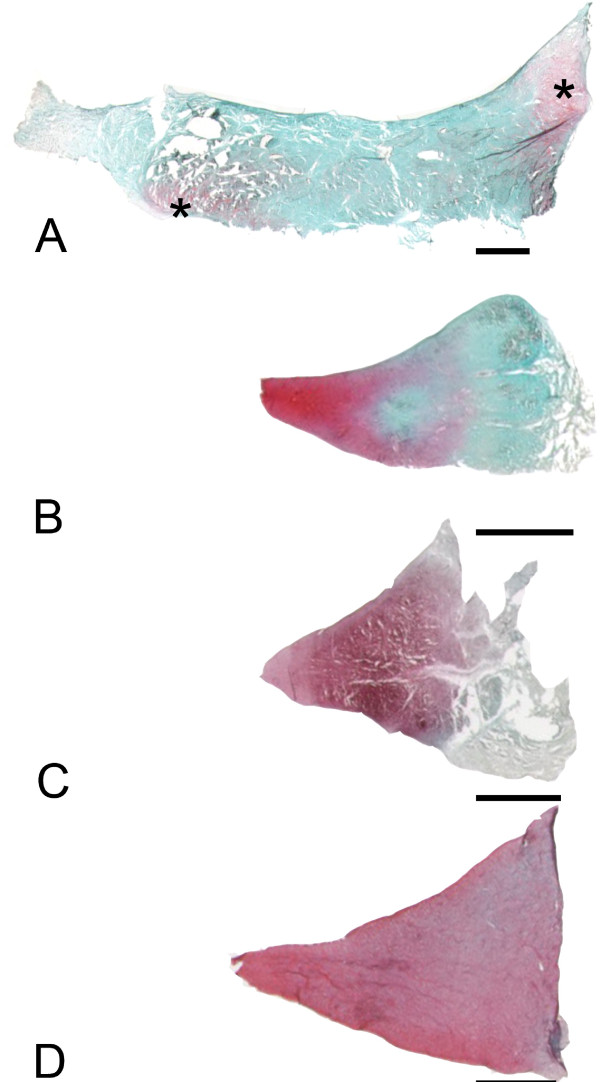
**Safranin O stained meniscus tissue from different species, all scale bars 3 mm. A)** Bovine meniscus with almost no prominent Safranin O staining. Only two localized regions (*) show some minor stain deposits. **B)** Ovine tissue, prominent red Safranin O staining at the inner third of the meniscus is visible. **C)** Human meniscus with two third of the inner tissue regions labeling red with Safranin O stain. Note the similarity in shape between ovine and human meniscus cross-sections. **D)** The porcine meniscus is labeling completely positive with Safranin O stain.

**Figure 6:**
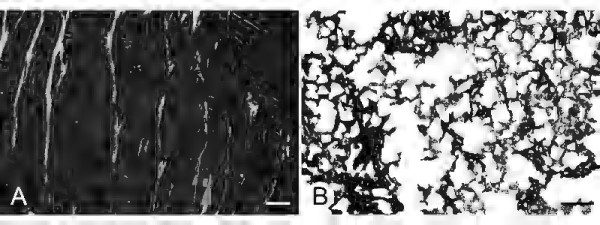
**Images of synthetic meniscus scaffolds. a)** CMI in polarized light exhibits a lose meshwork of interconnected fibers. Scale bar: 200 μm. **b)** Unstained Actifit section shows a granular structure without a clearly defined fiber orientation. Scale bar: 500 μm.

Bovine meniscus (Figure 5a) show almost no prominent Safranin O staining, whereas the porcine meniscus is labeling completely positive with Safranin O (Figure 5c). In contrast ovine tissue (Figure 5b) reveals a prominent red Safranin O staining at the inner third of the meniscus is. This labeling is comparable to the staining found in human mensicus samples (Figure 5c). There, two third of the inner tissue regions are labeling red with Safranin O stain. In addition, a similarity in shape between ovine and human meniscus cross-sections can be noticed The synthetic scaffolds show the acellular structure, with the Menaflex revealing similarities to the former tendon structure, whereas the Actifit shows the pure synthetic meshwork (Figure 6).

## Discussion

The objective of our study was to assess the biomechanical behavior of meniscal tissue from different large animals in comparison to normal and degenerated human meniscus tissue as well as two different artificial scaffolds. We found that the viscoelatisc properties of ovine meniscus samples show the highest congruence to human meniscus samples in our biomechanical setup and that the tested artificial scaffolds lack the typical meniscal properties at time zero.

Several different approaches have been described to evaluate the biomechanical properties of meniscus tissue: The creep indentation test characterizes the material properties with the aggregate modulus, Poisson’s ratio, permeability, and shear modulus [25]. Uniaxial tension tests are used for evaluation of meniscal attachment function after transplantation [27], tensile forces are used to evaluate suture function in meniscus repair techniques [28,29]. The ball indention test characterizes the viscoelastic properties of meniscal tissue including stiffness, residual force and compression behavior [9,30] and we chose this test also in our biomechanical setup as the cyclic testing mimics physiological loading conditions.

Joshi et al. [26] found that among monkey, canine, bovine and porcine samples the ovine menisci were most similar to human menisci. These data can only partially be compared to our work because a different biomechanical setup with uniaxial confined compression testing was used. Nevertheless, we could also confirm the biomechanical similarities between human and ovine samples in our own setup. Based on the work of Sweigart et al. who report biomechanical differences in the different regions of the porcine medial meniscus, we assured to harvest the meniscus samples from the same topographic region to avoid any bias [25].

Normal human and degenerative meniscus samples from the same region revealed significantly different stiffness values with lower values for the degenerative samples. In addition, we could show that human meniscus samples exhibited statistically significant differences in their viscoelastic behavior compared to the large animal menisci tested, except the ovine samples. As a consequence meniscus research focusing on the biomechanical properties should be performed using an ovine animal model.

Data about the biomechanical properties of Menaflex and Actifit before cellular ingrowth are sparse. The Actifit polyurethane scaffold shows a classic stress-strain response [31] and has slightly more viscoelastic behavior (higher compression and residual force), whereas its stiffness is less (not significant) than in the Menaflex scaffold. Both artificial constructs had significantly different viscoelastic properties in comparison to all tested human and animal samples.

Since the Menaflex samples are made from bovine collagen (mostly type I), its structure is well adapted to withstand tensile forces. During processing, water binding molecules like proteoglycans and glycosaminoglycans are almost entirely removed (Figure 5a), which explains the absence of viscoelastic behavior. In such scaffolds in-growing cells need to produce the specific extracellular matrix of meniscus, in order to gain “meniscal biomechanical properties” [32]. The Actifit as a fully artificial construct (Figure 5b) also lacks the viscoelastic properties of normal meniscus tissue. The polymer implants induced fibrous ingrowth with cartilaginous areas, which resembled neo-meniscal tissue. Though there have been little data in large weight-bearing models such as the ovine, it might be that the chondroprotective effects are more due to an improvement of the contact area than biologic effects [31]. A study by Welsing et al. [33] found that the porous polymer implants were fully integrated after 2 years in a dog model. Still there were no viable cells in all parts of the meniscus and their biomechanical properties were intermediate to normal meniscus and the scaffold before implantation. In contrast, Maher et al [34] could prove that the implantation of a polyurethane scaffold in a partial meniscectomy ovine model promotes tissue ingrowth without affecting the articulating cartilage. Transferring these results to daily clinical life, the artificial scaffolds must be protected from weight bearing until cellular in-growth and remodeling processes can occur and allow for maturation of the constructs and induce a gain of biomechanical strength. Considering their minor biomechanical properties at time zero partial weight bearing or even load removal of the operated leg for 6 weeks would be recommendable. Possibly, the cellularization of scaffolds before implantation might be beneficial in terms of biomechanical behavior and biocompatibility [13,30].

We believe that a large-animal model is necessary in meniscus experiments: First there are anatomical similarities between the human knee and the knee of a quadruped, like the sheep [35,36]. This includes the existence of cruciate ligaments, the menisci and the asymmetrical collateral ligaments with a bicondylar distal femur [37]. There is also a visible similarity when comparing cross-sectional shape of the midpart region of sheep and healthy human menisci (Figure 5). Second, viscoelastic meniscus behavior in a large animal model is more comparable to human menisci than it is in small animals due to the limited size of rabbit or rat meniscus. Third, small animals like rabbit or rat walk in a much more flexed knee position compared to humans or larger animals. Gait analysis in goats showed that these larger quadrupeds show similar knee flexion and full extension during walking or trotting and have comparable values to human patients [38]. Finally, Chevrier et al. [24] found that in the important histological terms of vascularization and cell density the ovine menisci were much more similar to human meniscus samples than rabbit menisci underlining our results.

Nevertheless, this study has several limitations. The tested meniscus samples were all taken from the intermediate part, so that no information about the anterior or posterior meniscal part can be made. In addition, the histological results are only descriptive- a systematic analysis is missing.

Due to the small size with difficult meniscus surgery in small animals we performed no biomechanical testing of small animals. However, there are some studies using the rabbit model for basic science research [39,40].

## Conclusions

In summary, we could show that from the large animals (bovine, ovine and porcine) the biomechanical and histological properties of the ovine meniscus samples revealed the highest similarity to human samples, in terms of stiffness, compression and residual force and that therefore the desirable animal model for meniscus research would be ovine meniscus tissue. Artificial scaffolds have inferior biomechanical properties and need to be protected from load transfer until remodeling processes might improve the viscoelastic properties.

## Competing interests

The authors declare that they have no competing interests.

## Authors’ contributions

All authors contributed in a significant way in the steps of processing the patient history as well as writing and editing the manuscript. GHS and TT conceived the idea for the study/publication, planning of the whole study and engaged in writing the manuscript. EGG and SD provided expertise in the biomechanical testing and statistics. CA and SM were responsible for the histological part. AB was responsible for collecting the human meniscus samples and preparing them for processing. ABI and SV gave advice throughout the project and reviewed the manuscript. RB was involved in the planning, the funding and the review process.

## Pre-publication history

The pre-publication history for this paper can be accessed here:

http://www.biomedcentral.com/1471-2474/14/324/prepub
